# Deep Learning Model for Predicting Lung Adenocarcinoma Recurrence from Whole Slide Images

**DOI:** 10.3390/cancers16173097

**Published:** 2024-09-06

**Authors:** Ziyu Su, Usman Afzaal, Shuo Niu, Margarita Munoz de Toro, Fei Xing, Jimmy Ruiz, Metin N. Gurcan, Wencheng Li, M. Khalid Khan Niazi

**Affiliations:** 1 Center for Artificial Intelligence Research, Wake Forest University School of Medicine, Winston-Salem, NC 27101, USA; uafzaal@wakehealth.edu (U.A.); mgurcan@wakehealth.edu (M.N.G.); mniazi@wakehealth.edu (M.K.K.N.); 2 Department of Pathology, Wake Forest University School of Medicine, Winston-Salem, NC 27101, USA; sniu@wakehealth.edu; 3 Department of Pathology, Stanford University School of Medicine, Stanford, CA 94305, USA; marmdt@stanford.edu; 4 Department of Cancer Biology, Wake Forest University School of Medicine, Winston-Salem, NC 27101, USA; fxing@wakehealth.edu; 5 Department of Hematology and Oncology, Wake Forest University School of Medicine, Winston-Salem, NC 27101, USA; jruiz@wakehealth.edu

**Keywords:** lung adenocarcinoma, recurrence prediction, whole slide images, histopathology, weakly supervised learning

## Abstract

**Simple Summary:**

This study introduces a deep learning model designed to predict the 5-year recurrence risk of lung adenocarcinoma based on histopathology images. Using a dataset of 189 patients with 455 histopathology slides, our model demonstrated superior performance in risk stratification, achieving a hazard ratio of 2.29 (95% CI: 1.69–3.09, *p* < 0.005). This outperforms several existing deep learning methods, showcasing the potential of deep learning in automatically predicting lung adenocarcinoma recurrence risk. The superior performance of this model underscores the potential for deep learning models to be integrated into clinical workflows for more accurate and automated risk assessment in lung adenocarcinoma. This could lead to more personalized treatment strategies and better patient outcomes.

**Abstract:**

Lung cancer is the leading cause of cancer-related death in the United States. Lung adenocarcinoma (LUAD) is one of the most common subtypes of lung cancer that can be treated with resection. While resection can be curative, there is a significant risk of recurrence, which necessitates close monitoring and additional treatment planning. Traditionally, microscopic evaluation of tumor grading in resected specimens is a standard pathologic practice that informs subsequent therapy and patient management. However, this approach is labor-intensive and subject to inter-observer variability. To address the challenge of accurately predicting recurrence, we propose a deep learning-based model to predict the 5-year recurrence of LUAD in patients following surgical resection. In our model, we introduce an innovative dual-attention architecture that significantly enhances computational efficiency. Our model demonstrates excellent performance in recurrent risk stratification, achieving a hazard ratio of 2.29 (95% CI: 1.69–3.09, *p* < 0.005), which outperforms several existing deep learning methods. This study contributes to ongoing efforts to use deep learning models for automatically learning histologic patterns from whole slide images (WSIs) and predicting LUAD recurrence risk, thereby improving the accuracy and efficiency of treatment decision making.

## 1. Introduction

Lung cancer is the leading cause of cancer deaths in the United States, surpassing the combined mortality of breast, colorectal, and prostate cancers [1]. The disease primarily manifests in two forms: non-small cell lung cancer (NSCLC), constituting 81% of cases, and small cell lung cancer (SCLC), comprising 14% of cases, with the remaining percentage representing other less common or unspecified types. NSCLC further subdivides into adenocarcinoma, the most prevalent histology, followed by squamous cell carcinoma and large cell carcinoma. While recent advancements in treatment have led to longer survival for lung cancer patients, the 5-year relative survival rate for lung cancer remains low, standing at 25% overall and 30% for NSCLC [2].

Lung adenocarcinoma (LUAD) is the most common histologic subtype of NSCLC treated with surgical resection. Microscopic evaluation of tumor grading is a standard pathologic practice and informs therapy and patient management across various organ systems [3,4]. However, there is a lack of consensus on a grading system for invasive LUAD. The 2015 WHO classification categorizes LUAD based on the predominant histologic pattern, dividing LUAD into three prognostic groups: low grade (lepidic predominant); intermediate grade (acinar or papillary predominant); and high grade (solid or micropapillary predominant) [5]. LUAD are histologically heterogeneous, displaying various combinations of patterns and proportions. Despite the acinar subtype being the most common (estimated at 40–50% of patients) when classified solely by the predominant pattern, it encompasses a wide range of prognoses [6,7,8]. 

Recently, the International Association for the Study of Lung Cancer (IASLC) pathology committee introduced a grading system for resected LUAD [9]. This model, based on pattern recognition, presents a novel prognostic grouping for LUAD that is reproducible across multiple data sets. It categorizes LUAD into three grades: Grade 1 (characterized by the lepidic pattern with less than 20% of high-grade pattern), Grade 2 (comprising the papillary or acinar pattern with less than 20% of high-grade pattern), and Grade 3 (encompassing any predominant pattern with 20% or more of high-grade pattern), where the high-grade pattern is defined as solid, micropapillary, or complex glandular pattern. This grading system has shown prognostic value for LUAD patients. Despite these advances in grading, accurately predicting recurrence remains a critical challenge. The variability in histologic patterns, particularly within common subtypes like acinar, complicates the assessment of recurrence risk. Understanding and predicting which patients are at higher risk for recurrence after surgical resection is essential for improving long-term outcomes.

In addition to a histologic grading system, several approaches have been proposed to predict the likelihood of recurrence within five years after surgery. One emerging method is the use of circulating tumor DNA (ctDNA), which has been shown to be a valuable biomarker for cancer diagnosis, treatment selection, and outcome prediction [10]. Its efficacy may be limited, especially when the DNA quantity is minimal, leading to potential failures in accurately predicting recurrence in clinical settings [11]. Furthermore, the predictive reliability of ctDNA for recurrence is not yet fully established; therefore, its use is currently limited to academic settings. Gene expression profiling is another critical method used in predicting recurrence in LUAD. It involves analyzing the patterns of gene expression to provide insights into the behavior of cancer, including its likelihood of recurrence. Several specific gene expression profiling tests are used for this purpose, including Oncotype DX, MammaPrint, PAM50 (Prosigna), CancerType ID, and FoundationOne CDx. Nevertheless, these genetic tests are subject to high costs and limited availability. Studies have reported that the utility of these genetic tests is relatively low among underrepresented communities, exacerbating disparities in access to personalized cancer care and potentially leading to unequal treatment outcomes [12,13]. 

Deep learning has been widely applied to predict cancer patients’ outcomes from digital pathology images, with survival and recurrence outcome prediction being among the most common applications [14,15,16,17]. Compared with manual analysis, deep learning models can automatically learn prognostic image features without introducing human bias. Over the past five years, deep multiple-instance learning (MIL) models have been widely adopted for various pathology analysis tasks, including metastasis prediction [18,19], cancer subtyping [20,21], survival prediction [22,23], etc. One key advantage of MIL is its ability to learn from high-resolution WSIs that are labeled or associated with clinical outcomes, without needing pixel-level annotations [24]. However, there are limited studies utilizing MIL models to predict the recurrence of LUAD patients from WSIs. To facilitate the prognosis of LUAD, we performed this study with a dataset collected from stage I to III LUAD patients. Specifically, we propose a deep learning model named dual-attention-based multiple-instance learning (DAMIL) to predict the 5-year risk of recurrence of LUAD patients after surgery.

## 2. Materials and Methods

### 2.1. Datasets

In our retrospective study, we included 195 patients diagnosed with stage I to III LUAD who underwent resection at Wake Forest Baptist Comprehensive Cancer Center (WFBCCC) between 2008 and 2015. All patients were followed up for a minimum of five years. Our study exclusively included invasive adenocarcinomas that align with the new grading system proposed by the IASLC. We did not include in situ or minimally invasive adenocarcinomas, given their nearly 100% five-year survival rate. Additionally, mucinous adenocarcinomas were excluded, as they are not part of the new grading system. The tumor resection slides were stained with hematoxylin and eosin (H&E) and scanned into whole slide images (WSI) using an Olympus VS200 Slide Scanner. We excluded slides that failed to go through our image preprocessing pipeline. As a result, 189 patients with 455 WSIs were analyzed in our study. Detailed characteristics of our dataset are depicted in Table 1. This study was approved by the Institutional Review Board of Wake Forest University (Approval no. IRB00074626) and was performed in accordance with the Declaration of Helsinki. To feed the WSIs into the deep learning model, we cropped the WSIs into 896 × 896 pixel patches under 40× magnification.

### 2.2. Tumor Bulk Segmentation

Tumor bulks are the primary tumor regions on the resections. To enhance the focus of deep learning models on the most critical information, it is standard practice to perform recurrence predictions on these tumor bulk areas within the slide [25,26]. Thus, as the first step of our model, we performed tumor bulk segmentation to remove the irrelevant (normal) tissue regions from WSIs. 

We approached tumor bulk segmentation as a patch classification problem, dividing the WSIs into small image patches and classifying each patch as either tumor or normal tissue. Initially, we extracted the foreground (i.e., tissue regions) from the WSIs using a color thresholding method [27]. 

**Figure 1:**
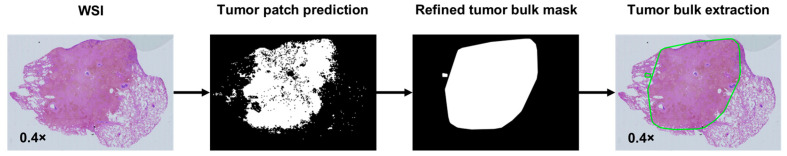
Overview of our tumor bulk segmentation pipeline, including tumor patch prediction, tumor bulk mask refinement, and tumor bulk extraction.

We then trained a deep learning-based patch classifier model for tumor/normal classification. For this, our experienced pathologist annotated the tumor bulk regions from 20 WSIs. We randomly cropped patches from these annotated tumor bulk regions and normal regions to train our patch classifier. Using the trained patch classifier, we applied it to all the foreground patches from each WSI, mapping the results into a binary mask for each WSI. To refine this tumor bulk mask, we employed a series of morphological transformations, including binary closing, small object removal, and convex hull operations. The overview of tumor bulk segmentation is depicted in Figure 1.

### 2.3. Multiple-Instance Learning for LUAD Recurrence Prediction

The prediction of cancer recurrence is a critical aspect of patient management, especially for conditions like LUAD, where timely and accurate prognostic information can significantly impact treatment decisions and patient outcomes. Traditional methods of predicting recurrence rely heavily on manual assessment of histological patterns, which can be subjective and prone to inter-observer variability. This approach also imposes a substantial burden on pathologists due to tumor histologic heterogeneity, which requires extensive microscopic examination. In response to these challenges, we aim to develop a deep learning model that leverages WSIs to predict the 5-year recurrence of LUAD patients based on the tumor bulk region.

Previously, several MIL models have been proposed for cancer prognosis [14,28,29]. However, most of these models require both histopathology and genomic data, which can be challenging to obtain and integrate. Moreover, comprehensive genomic tests are not always performed in resected early-stage LUAD, further limiting the applicability of these models. In our previous study, we proposed a weakly supervised learning model that can accurately risk stratify HR+/HER2− breast cancer patients based solely on histopathology slides [15]. Nevertheless, this model is not parameter-efficient due to its multi-branch cross-attention design, potentially limiting its convergence performance when trained on small datasets.

For predicting the recurrence of LUAD, we estimate the likelihood of recurrence within five years based on the analysis of segmented tumor bulk regions. For simplicity, we denote the category of recurrence within five years as RC and the category of no recurrence as NRC. To achieve this, we propose a dual-attention-based multiple-instance learning (DAMIL) model that is more powerful and computationally efficient than our previous recurrence prediction model [15]. 

The first and also standard step of an MIL involves cropping WSIs into patches and encoding these patches into embeddings, thereby reducing the WSIs into vector representations that are scalable for computational resources. We employ a histopathology-specific model to encode each 896 × 896 patch into a 768-dimensional embedding [30]. Embeddings from the same WSI are grouped to form a bag, with each bag labeled as either RC or NRC, depending on whether the patient experienced recurrence within five years. The MIL model is then responsible for aggregating the patch embeddings within each bag and mapping them to a bag-level (WSI-level) prediction.

The primary idea of our model is to extract a set of representative patch embeddings from each category, namely the key sets. Then, our model correlates the key sets with the input patch embeddings using a cross-attention neural network. Therefore, our first step is categorical representation learning (CRL) [15,18]. In a nutshell, given a matrix of all patch embeddings from a WSI, our CRL method extracts the high statistical leverage patch embeddings as the representation of the WSI. All the patches are embedded using a pre-trained histopathology image encoder [30]. All extracted embeddings from the RC and NRC WSIs in the training set are concatenated as a key set $K=\left[k_{1},k_{2},\ldots ,k_{\tau }\right]\in {\mathbb{R}}^{D\times \tau }$, where *k* indicates the extracted patch embedding, D indicates the embedding dimension, and τ indicates the total number of embeddings in a key set. The details of this method can be found in our previous studies [15,18]. Unlike our previous method, where we built separate key sets for different categories, here we join all embeddings into one key set to build a more parameter-efficient model.

In the next step, we develop an MIL model for recurrence prediction. Our MIL model utilizes a dual-attention architecture. The first component of this model is a cross-attention neural network. Cross-attention can be used to introduce external guidance into the model to be fused with the input data [31,32]. In our case, the external guidance is the key set that represents the typical patch embeddings for RC and NRC categories. Let’s assume a key set $K\in {\mathbb{R}}^{D\times \tau }$ and an input WSI containing *n* patch embeddings $Q=\left[q_{1},\;q_{2},\;\ldots ,\;q_{n}\right]\in {\mathbb{R}}^{D\times n}$. We perform the cross-attention as a transformer decoder does [33]:

(1) $$crossAttn\left(\tilde{K},\tilde{Q},\tilde{V}\right):H=softmax\left(\frac{{\tilde{K}}^{T}\tilde{Q}}{\sqrt{D_{h}}}\right){\tilde{V}}^{T}$$

where $\tilde{K}\in {\mathbb{R}}^{D_{h}\times \tau }$ is the embedded key set K, $\tilde{Q}\in {\mathbb{R}}^{D_{h}\times n}$ and $\tilde{V}\in {\mathbb{R}}^{D_{h}\times n}$ are the embedding of input Q, $H\in {\mathbb{R}}^{\tau \times D_{h}}$ is the output of the cross-attention operation, and $D_{h}$ is the dimension of the hidden layers for this model.

**Figure 2:**
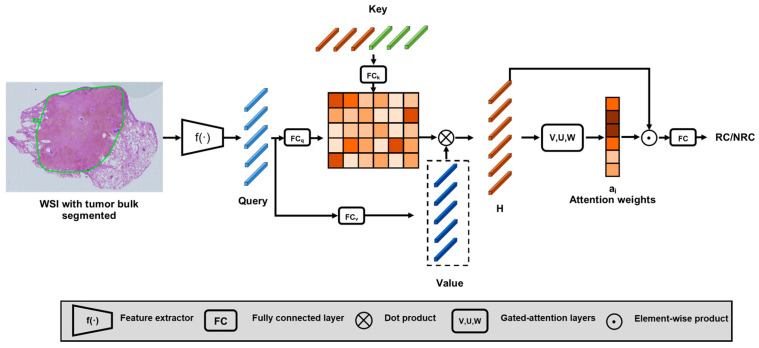
Overview of the DAMIL architecture. The tumor bulk region from a WSI is extracted and cropped into patches during the preprocessing step. All patches are further encoded into vectors by a feature extractor model. The encoded vectors go through a cross-attention module followed by a gated-attention module. The final representation is used for RC/NRC prediction.

As the second attention component of our MIL model, we employ a gated-attention neural network to highlight the crucial embeddings from the output of the cross-attention neural network [34]. It is performed in this manner:

(2) $$a_{i}=\frac{\exp\left\{W\left(\tanh\left(Vh_{i}^{T}\right)⊙sigm\left(Uh_{i}^{T}\right)\right)\right\}}{\sum_{j}^{\tau }\exp\left\{W\left(\tanh\left(Vh_{j}^{T}\right)⊙sigm\left(Uh_{j}^{T}\right)\right)\right\}}\;$$

(3) $$z=\overset{\tau }{\underset{i}{\sum }}a_{i}h_{i}$$

where V and $U\in {\mathbb{R}}^{L\times D_{h}}$, $W\in {\mathbb{R}}^{1\times L}$ are fully connected neural networks. $h_{i}\in {\mathbb{R}}^{1\times D_{h}}$ denotes embeddings in H that are scaled by the attention weights $a_{i}\in \mathbb{R}$ and summed to the slide-level representation $z\in {\mathbb{R}}^{1\times D_{h}}$. In this way, our DAMIL model has embedded and summarized a high-resolution WSI into a single vector as a slide-level representation. Finally, we apply a fully connection network on to *z* to make a slide-level prediction. We have named our model DAMIL, which stands for dual-attention multiple-instance learning. The overview is depicted in Figure 2.

### 2.4. Implementation Details

For WSI preprocessing, we first applied color thresholding on the thumbnails of WSIs to extract the tissue regions [27]. These tissue regions were then cropped into 896 × 896 patches at 40× magnification. After filtering out patches from the tumor bulk region, we encoded each patch into patch embeddings of 768-dimension by CTransPath [30], a histopathology-specific foundation model for patch encoding. Before encoding, all patches were resized into 224 × 224 to match the input size of CTransPath. In a nutshell, this 224 × 224 patch corresponded to a 10× magnification of an 896 × 896 patch cropped at 40× magnification. To be noticed, we cropped patches at 40× magnification since we wanted to resize the patches from their earliest-scanned version, which was typically under 40×, so that all the patches were consistent in scale across different WSIs.

We trained the proposed model using the Adam optimizer [35] with a learning rate of 0.00008 and a weight decay of 0.00001. The training process began with 5 initial epochs, followed by early stopping if the validation loss did not decrease for 5 consecutive epochs. The maximum training duration was capped at 50 epochs. Due to the varying bag sizes of different WSIs, we used a batch size of one, which is common in MIL models for WSI analysis since PyTorch requires uniform data shapes within each batch. To address class imbalance during training, we employed a weighted sampling strategy that assigned a higher sampling rate to RC cases compared with NRC cases. This approach helped mitigate the imbalance by ensuring that recurrence cases were more frequently included during each epoch.

### 2.5. Experiment Approach

We divided the WSIs into RC/NRC categories based on whether recurrence occurred within five years. We performed a stratified five-fold cross-validation on our dataset. The training/validation/testing splitting was done at patient-level to avoid data leakage issues. The dataset distribution for cross-validation is summarized in Table 2. 

We performed a survival analysis by fitting a univariate Cox proportional-hazard model to correlate our binary classification results with patients’ recurrence-free durations over five years. The results were evaluated using the hazard ratio. Additionally, we also reported our classification performance using metrics such as the area under the receiver operating characteristic curve (AUROC), accuracy, specificity, and sensitivity. 

## 3. Results

In this section, we present our experimental results for 5-year recurrence prediction. We mainly compare our model with two existing deep learning models, CLAM [27] and DeepODX [15]. CLAM is a MIL model that is widely used in various tasks of WSI analysis. DeepODX, introduced in our previous study, is a breast cancer risk stratification model that has demonstrated excellent accuracy in predicting patient recurrence risk. In Section 3.1, we demonstrate DAMIL’s recurrence stratification performance by a survival analysis. In Section 3.2, we show DAMIL’s binary classification performance for exact 5-year recurrence. In Section 3.3, we compare DAMIL with our previous model to show the computational efficiency of our new model.

### 3.1. Survival Analysis for 5-Year Recurrence

**Figure 3:**
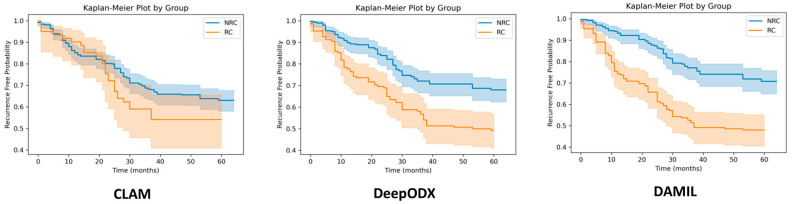
Kaplan–Meier plots for recurrence-free probability for 5-year follow-up.

Table 3 shows the survival analysis performance of our DAMIL and comparison methods. The proposed model achieved a hazard ratio of 2.29 (95% CI: 1.69–3.09, *p* < 0.005), which outperforms the comparison MIL models. Additionally, we visualized the Kaplan–Meier plots in Figure 3. It further shows that the proposed model can clearly stratify the patients for their recurrence risk.

### 3.2. Binary Classification Performance for 5-Year Recurrence

Table 4 reports the 5-year recurrence classification performance of the proposed model and comparison MIL methods. It exhibits a reasonable and stable performance in determining patients’ 5-year recurrence based solely on histopathology slides.

Table 5 presents the functional loss of the DAMIL model across the training, validation, and testing sets. The results indicate a gradual degradation in performance from the training set to the testing set, as reflected by an increase in loss. This degradation is within expected limits and can likely be attributed to the small dataset size. Enhancing the dataset by incorporating additional WSIs could help address this issue and improve the model’s generalization ability.

### 3.3. Computational Analysis

Table 6 reports the computational analysis results comparing our dual-attention-based approach with DeepODX, which uses a multi-branch cross-attention architecture. The analysis indicates that our updated model is more computationally efficient. This enhancement is particularly important in computational pathology, where small datasets are often encountered. By reducing the model’s parameters, we have created a more lightweight architecture that helps mitigate the risk of overfitting.

## 4. Discussion and Conclusions

In this study, we proposed a deep learning model for predicting 5-year recurrence in patients with surgically resected LUAD based on tumor bulk regions in WSIs. According to the Lung and Bronchus Cancer Stat Facts from the National Cancer Institute, the 5-year relative survival rate for patients with potentially resectable lung cancer ranges from 35.9% (regional tumor) to 63.7% (localized tumor). This statistic highlights the critical need for improved predictive models and refined follow-up and treatment strategies for patients with potentially resectable lung cancer. Our project aims to enhance the accuracy of recurrence risk stratification, which could lead to earlier interventions and more personalized treatment plans. 

In the literature, several MIL models are proposed for predicting cancer recurrence/survival from WSIs [14,28,29]. Nevertheless, most of them utilize multi-modal data including genomic data, imaging, and clinical information, which increases the complexity of acquiring complete data. Therefore, we proposed DAMIL, which employs dual-attention architecture for predicting LUAD recurrence using WSI data only. Several existing MIL models have different dual-attention design. Existing dual-attention MIL models follow different strategies. A common approach [36,37] is to first apply spatial attention to each instance, such as a patch from an MRI or a slice from a CT scan, embedding the images into feature vectors. Then, instance-wise attention is applied to aggregate the instances into a single-feature vector. Another method by Xu et al. [38] performs spatial-wise and instance-wise attention in parallel to capture both global and local features. However, these approaches often require feeding raw images as instances into the MIL model, which can cause GPU memory issues, especially with WSIs that can be divided into thousands of patches.

In contrast, Chen et al. proposed applying dual attention directly on patch embeddings, which reduces memory usage [22]. Their model employs cross-attention to integrate patch embeddings with genomic features, followed by instance-wise attention for both patches and genomics separately. While effective, this approach requires paired WSI-genomic data for each patient, which is challenging to obtain. In our proposed DAMIL, instead of using cross-attention to combine multi-modal data, we leverage it to aggregate patch embeddings based on different categorical representations derived from the WSIs themselves. This allows us to directly incorporate categorical information related to patient recurrence into the patch embeddings. Finally, we apply gated attention to combine all learned features for accurate WSI-level prediction. This approach distinguishes our model from previous dual-attention MIL models and demonstrates promising performance in LUAD recurrence prediction.

Table 3 demonstrates that our proposed DAMIL significantly outperforms the comparison MIL models, including CLAM and DeepODX, in predicting the 5-year recurrence of LUAD patients. The DAMIL model achieved a hazard ratio of 2.29 (95% CI: 1.69–3.09, *p* < 0.005), indicating a strong ability to differentiate between high and low-risk patients. The Kaplan–Meier plots in Figure 3 further illustrate the effectiveness of our model in risk- stratifying patients, providing clear separation between the risk groups. This performance underscores the robustness and reliability of the DAMIL model.

The results in Table 4 highlight the superior performance of our proposed DAMIL model in predicting the 5-year recurrence of LUAD patients. Compared with the existing MIL models, CLAM and DeepODX, our DAMIL model achieves higher metrics across the board: an AUROC of 64.9 ± 1.2, accuracy of 63.5 ± 3.1, specificity of 69.3 ± 6.6, and sensitivity of 53.0 ± 7.9. These results indicate that the DAMIL model not only improves overall classification performance but also offers a better balance between specificity and sensitivity.

The promising performance of our DAMIL model in recurrence risk stratification holds significant clinical implications. By achieving a high hazard ratio and outperforming several existing deep learning methods, our model demonstrates its potential to enhance the accuracy of predicting 5-year recurrence in patients with resectable LUAD. The ability to accurately stratify recurrence risk allows clinicians to identify high-risk patients who may benefit from more aggressive treatment and closer monitoring. Currently, tumor staging and grading are the major components in assessing cancer prognosis; however, they are not without limitations, since the majority of surgically resectable LUADs are early stage, and tumor grading can sometimes be imprecise due to interobserver variability and inherent biological heterogeneity. Incorporating our model into the traditional methods will further refine its predictive power, allowing for a more precise risk assessment. This proactive approach could potentially improve patient outcomes by addressing recurrence early and reducing mortality rates. Moreover, the application of DAMIL in clinical practice could potentially optimize resource allocation. 

By accurately predicting the biology of LUAD and the risk of recurrence, we can seek to study the impact of standard or novel therapies in the future. Additionally, our model’s success in outperforming existing methods suggests that integrating dual-attention mechanisms with multiple instance learning can provide a more nuanced understanding of tumor heterogeneity. This advance in modeling could inspire further research into similar approaches for other cancer types, thereby broadening the impact of our findings. Finally, the ability to automatically learn and utilize prognostic features from WSIs without human bias offers significant advantages in clinical practice. By reducing reliance on subjective interpretations, our model minimizes potential inconsistencies and errors associated with manual assessment. This not only enhances the objectivity of prognostic evaluations but also alleviates the workload on pathologists.

While our DAMIL model shows strong performance in predicting 5-year recurrence, several limitations must be noted. The study’s small dataset may limit the model’s generalizability and increase the risk of overfitting. Additionally, the dataset is imbalanced, with few recurrence cases, which may affect the model’s sensitivity and overall predictive accuracy. Clinically, this imbalance could impact the model’s utility in settings where recurrence is rare but critical to identify. In addition, the model’s reliance solely on histopathology slides, without multi-modal data integration, may restrict its potential. Combining histopathology with genomic and other data types could enhance predictive performance and provide a more comprehensive risk assessment. Finally, our study was developed and validated using data from a single hospital, limiting our ability to assess the model’s generalizability across different institutions. Training and testing the model on data from multiple hospitals and diverse communities will be essential to evaluating its robustness and applicability in broader clinical settings.

In future work, we plan to include more patients, particularly recurrence cases, from multiple hospitals to enhance both training and validation. Our data collection will also focus on more diverse patient cohorts to evaluate the model’s performance across different populations and clinical environments. To improve the generalizability of our model, we also intend to apply stain normalization on WSIs in our future multi-center study to address stain variability across different institutions. In addition, we would include more demographic and pathologic features such as smoking status, tumor staging/grading, and genomic profile to enhance our model with a multi-model approach.

This study proposed a deep learning-based computational pathology model to automatically predict 5-year recurrences in LUAD patients based on H&E-stained WSIs. Our model exhibits excellent 5-year recurrence risk stratification, with a hazard ratio of 2.29 (95% CI: 1.69–3.09, *p* < 0.005). Moreover, our model is more computationally efficient compared with the existing method, which can alleviate the risk of overfitting. In conclusion, this model shows potential for integration into clinical practice as a prognostic tool.

## Figures and Tables

**Table 1:** Dataset statistics of patients in this study.

	All	Recurrence in 5-yr	No Recurrence in 5-yr	*p*-Value
**Age**				0.964
Median [Min, Max]	63.5 [39, 88]	66 [39, 84]	63 [43, 88]	
**Sex**				0.0018
M	77	39	38	
F	107	30	77	
**Race**				0.0008
White	149	57	92	
African American	23	7	16	
American Indian or Alaska Native	6	3	3	
Hispanic	3	1	2	
Native Hawaiian or Other Pacific Islander	1	0	1	
Asian	1	0	1	
Other	1	1	0	
**IASLC grade**				<0.0001
G1	24 (12.7%)	1 (1.4%)	23 (19.5%)	
G2	53 (28%)	9 (12.7%)	44 (37.3%)	
G3	112 (59.3%)	61 (85.9%)	51 (43.2%)	
**AJCC stage**				<0.0001
IA	90 (47.6%)	21 (29.6%)	68 (58.1%)	
IB	41 (21.7%)	15 (21.1%)	26 (22.2%)	
IIA	4 (2.1%)	3 (4.2%)	1 (0.9%)	
IIB	37 (19.6%)	20 (28.2%)	17 (14.5%)	
IIIA	12 (6.3%)	9 (12.7%)	3 (2.6%)	
IIIB	3 (1.6%)	3 (4.2%)	0	
IVA	2 (1.1%)	0	2 (1.7%)	

**Table 2:** Data distribution of each cross-validation fold.

	RC (*n* of WSIs/Patients)	NRC (*n* of WSIs/Patients)
Training	127/51	179/75
Validation	14/6	37/19
Testing	29/14	59/24

**Table 3:** Stratification performance based on Cox proportional-hazard model.

	Hazard Ratio
CLAM [27]	1.33 (95% CI: 0.89–2.00, *p* = 0.17)
DeepODX [15]	1.88 (95% CI: 1.39–2.55, *p* < 0.005)
DAMIL	**2.29 (95% CI: 1.69–3.09, *p* < 0.005)**

**Table 4:** Five-year recurrence classification performance. Performance is reported with average ± standard deviation.

	AUROC	Accuracy	Specificity	Sensitivity
CLAM [27]	60.2 ± 10.6	60.7 ± 5.4	87.9 ± 11.7	16.9 ± 15.3
DeepODX [15]	61.2 ± 6.2	62.3 ± 3.5	73.6 ± 6.1	44.0 ± 5.4
DAMIL	**64.9 ± 1.2**	**63.5 ± 3.1**	69.3 ± 6.6	**53.0 ± 7.9**

**Table 5:** Functional loss across training, validation, and testing sets for DAMIL. Performance is reported with average ± standard deviation.

	Loss
Training set	0.466 ± 0.049
Validation set	0.628 ± 0.050
Testing set	0.674 ± 0.037

**Table 6:** Computational analysis comparing DAMIL against DeepODX. The computation is based on an input bag with shape (1, 120, 1024) under the evaluation mode.

	FLOPs	Param
DeepODX [15]	0.43G	3.15M
DAMIL	0.31G	2.10M

## Data Availability

Our code is available at https://github.com/cialab/lung-recurrence-prediction (accessed on 1 September 2024).
